# P-962. Designing ID Elective Goal and Learning Objectives Based On Need Assessment From Learners

**DOI:** 10.1093/ofid/ofae631.1152

**Published:** 2025-01-29

**Authors:** Jungwon Yoon, David S Hatem

**Affiliations:** UMass Chan Medical School, Worcester, Massachusetts; University of Massachusetts, Worcester, Massachusetts

## Abstract

**Background:**

Comprehensive Infectious disease (ID) education is indispensable for clinicians, given challenges posed by antimicrobial resistance, pandemics, expanding immunocompromised population, and the effect of climate change. ID fellowship applications have failed to match the demand for training programs. While numerous factors contribute to the prevailing challenges in ID workforce, enhancing clinical education in ID holds promise for bridging this gap.

Need Assessment Survey Results

**Figure:**
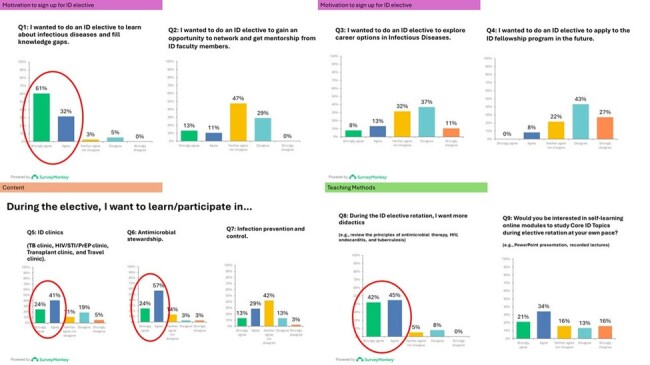

**Methods:**

An anonymous survey was conducted to understand the reasons learners in undergraduate (UME) and graduate medical education (GME) choose ID electives, what and how they want to learn during the rotation. In the academic year 2023, there were 59 GME and 10 UME learners enrolled in ID elective at our institution. 38 responded to the survey (55% response rate).

**Results:**

The survey showed 93% of respondents opted for electives to augment their knowledge. 24% sought networking and mentorship from faculty members, and 21% wanted to explore career options in ID. However, only 8% plan to apply to ID fellowship programs. Learners expressed interest in exploring outpatient ID clinics (63%), antimicrobial stewardship initiatives (81%), and infection control activities (42%), alongside inpatient consultation services. Moreover, a majority (87%) expressed a desire for structured didactics.

**Conclusion:**

While most pursue ID electives to address knowledge gaps, 21% of learners seem to be in an exploratory phase, seeking mentorship and contemplating a career in ID. ID electives have potential to serve as a platform for early trainees and students to explore their interests in the field. To optimize their experience, we could provide interactive didactics and diversifying clinical activities such as antimicrobial stewardship initiatives, and subspecialty clinics. Such initiatives not only provide learners with comprehensive insights into the field but also foster a deeper appreciation for the multifaceted nature of infectious diseases. Limitations from this study include small sample size and low response rate which impact generalizability of this finding. Conducting longitudinal surveys and evaluations with elective learners could provide further insights into how to improve the curriculum and better meet the needs of participants.

**Disclosures:**

**All Authors**: No reported disclosures

